# Relationship of placental edge thickness and cervical length to gestational age at delivery in patients with placenta previa

**DOI:** 10.12669/pjms.38.5.5097

**Published:** 2022

**Authors:** Wajeeha Syed, Nazia Liaqat, Ghazala Naseeb, Sumbal Mahmood Khattak

**Affiliations:** 1 Dr. Wajeeha Syed, FCPS Obstetrics & Gynecology. Assistant Professor, Department of Obstetrics & Gynecology, Medical Teaching Institute Lady Reading Hospital Peshawar, KPK, Pakistan; 2 Dr. Nazia Liaqat, FCPS Obstetrics & Gynecology. Assistant Professor, Department of Obstetrics & Gynecology, Medical Teaching Institute Lady Reading Hospital Peshawar, KPK, Pakistan; 3 Dr. Ghazala Naseeb, MBBS. Training Medical Officer, Department of Obstetrics & Gynecology, Medical Teaching Institute Lady Reading Hospital Peshawar, KPK, Pakistan; 4 Dr. Sumbal Mahmood Khattak, MBBS. Training Medical Officer, Department of Obstetrics & Gynaecology, Medical Teaching Institute Lady Reading Hospital Peshawar, KPK, Pakistan

**Keywords:** Placental edge thickness, Cervical length, Placenta previa, Preterm delivery

## Abstract

**Background & Objective:**

Placenta previa and its accompanying fetomaternal complications are increasing day by day because of globally increasing rates of cesarean deliveries, trends of assisted reproductive techniques, and delaying pregnancies to later ages. Placenta previa is an important contributor to iatrogenic and mostly emergent preterm deliveries, which add to increased neonatal morbidity and mortality. The predictors of preterm deliveries can help clinician make necessary preparations for optimal fetomaternal outcome. The aim of the current study was to determine relationship of placental edge thickness and cervical length with gestation at delivery in patients having placenta previa.

**Methods:**

It was a descriptive case series study conducted in the department of obstetrics and gynecology, Medical Teaching Institute Lady Reading Hospital Peshawar from January 2020 till January 2021 .Seventy five patients fulfilled the inclusion criteria and were included in the study. Already diagnosed cases of placenta previa, with singleton alive pregnancies and gestation of 28 weeks or more were enrolled. Trans vaginal ultrasound was done to determine placental edge thickness and cervical length. Patients data on gestation at delivery were collected from hospital records.

**Results:**

A significant negative correlation between the placental edge thickness and gestational age at delivery was seen (r= -0.566, P= 0.001). The correlation between length of cervical canal and gestation at delivery was positive (r= 0.362, P=0.001). Also thick placental edge of > 2cm had significant association with birth before 37 weeks(P=0.023). A short cervix of less than 2.5 cm had also statistically significant association with birth before 37 weeks (P=0.022).

**Conclusion:**

There is linear inverse relationship of placental edge thickness with gestation at delivery. The relationship of cervical length with gestation is positive linear. Patients with thick placental edge are more likely to deliver preterm than those having thin placental edge.

## INTRODUCTION

Placenta previa is a potential killer obstetrical condition, the rate of which is progressively increasing because of escalating rates of cesarean section, increased maternal age and practice of assisted reproductive techniques.^1^ Maternal smoking^2^ and cocaine use^3^ are also positively associated with placenta previa. It is a defined risk factor for preterm birth.^4^,^5^ Research has proposed that a certain degree of unprovoked placental parting do occur in such cases due to development of lower uterine segment and cervical dilatation ending up in hemorrhage and need for preterm delivery.^6^

Prenatal assessment of predictors for preterm delivery can improve preparation and management; like admission to hospital, blood arrangement, availability of beds in neonatal units, and presence multidisciplinary consultant led care. Royal college of obstetricians and gynecologists guideline 27 a recommends that cervical length measurement by transvaginal route facilitates management choices in asymptomatic women with placenta previa.^7^ Ghi T et al stated that women with central placenta previa and cervix of less than 31mm are 16 times more likely to end up on preterm emergency cesarean section because of haemorrhage.^8^

Thick placental edge is thought to be responsible for late preterm births i.e. birth at 36 weeks of pregnancy. Mustafa M et al in their study have recommended that placental edge thickness and cervical length measurement by transvaginal route should be a routine measurement in third trimester scan in patients with complete placenta previa.^9^

Incidence of placenta previa at term is 1.9-2.3% in local studies i.e. 19/1000 to 23/1000 deliveries.^10^,^11^ Keeping in view the high morbidity and mortality associated with preterm delivery in our setup, with limited neonatal intensive care units the current study was planned. As per our knowledge no local study has been done on this topic. We evaluated length of cervical canal and placental edge thickness in patients with placenta previa after 28 weeks with the intent to detect patients at risk of preterm delivery. The rationale was to incorporate these measurements into departmental protocol for management of placenta previa and to open window for further research on the subject.

## METHODS

This descriptive case series study was conducted in the department of obstetrics and gynecology, Medical Teaching Institute Lady Reading Hospital Peshawar from January 2020 till January 2021. Ethical approval was taken from hospital ethical review board (Ref# 543 dated 28-07-2020). Informed consents were taken from all the eligible included patients. Non probability consecutive sampling was done.

Already diagnosed cases of placenta previa, with singleton alive pregnancies at or beyond 28 weeks’ gestation, were included in the study. Following patients were excluded: patients with morbidly adherent placentas, hemodynamic instability, co-existing placental abruption, fetal anomalies, medical co morbidities like diabetes, hypertension, and patients having previous history of preterm deliveries.

Placental edge thickness and cervical length measurements were taken in all included patients using transvaginal ultrasound by the skilled expert sonologist. Measurement of placental edge thickness of non- central placentas was taken as the maximum measurement in the plane perpendicular to the long axis of placenta, within 2 cm of the lead point. For central placentas the thickness of the portion of placenta directly overlying the internal cervical os was taken. Patients data about the primary outcome measures of gestational age at delivery and secondary outcome measures of neonatal birth weight and Apgar score along with all other relevant information including patient’s age, parity, placental edge thickness, cervical length, gestation at the time of delivery, birth weight of neonate, Apgar score were noted on a pre designed proforma. Sample size was calculated by using online Open Epi, sample size calculator using 95% Confidence interval, Proportion of placenta previa as 0.5%, Margin of Error 2.3 and power of test as 80%.^12^ Data was analyzed on SPPSS-version 25. Descriptive statistics were determined for variables. Mean and standard deviations were calculated for continuous variables and frequency percentages were determined for categorical variables. Correlation coefficients with scatter plots were designed to determine relationship of placental edge thickness and cervical length with gestation at delivery. P -values were computed to measure significance of association between placental edge thickness and preterm delivery by applying chi-square test. A p-value of ≤ 0.05 was taken as significant (two tailed test).

## RESULTS

Out of total 75 patients, there were 10 (13.3%) primiparas and 65(85.3%) multiparous women. A total of 52(69.3%) patients delivered before 37 completed weeks and 23(30.07%) patients delivered at or beyond 37 weeks of gestation. The mean age of patients was 28.44 ± 3.95 and the mean gestational age at delivery was 36.40 ± 2.07.

**Fig.1:**
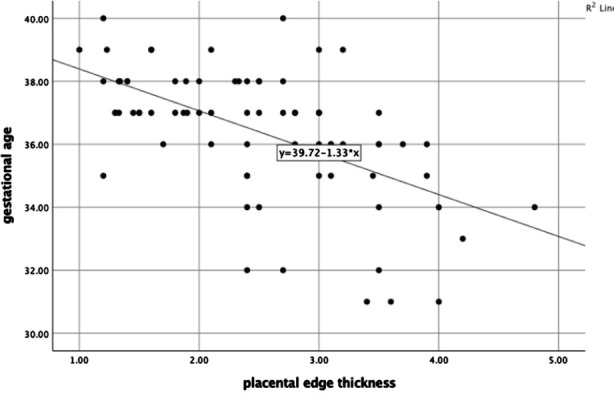
Scatter plot of correlation between placental edge thickness and gestational age at delivery.

**Fig.2:**
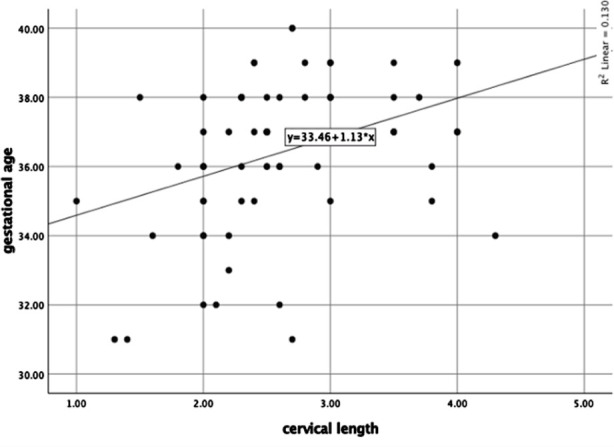
Scatter plot of correlation between cervical length and gestational age at delivery.

Among the fetal outcomes 11 (14.7%) had Apgar score less than 7, while 64(85.3%) had Apgar score more than 7. The mean weight of newborn was 2.82 ± 0.47. (Table-I.) There was a significant negative correlation between the placental edge thickness and gestational age at delivery, with P = 0.001 and Pearson correlation coefficient r= -0.566. (Fig.1). There was a significant positive correlation between cervical length and gestation at delivery. P=0.001, r= 0.361. (Fig.2).

**Table I:** Descriptive Statistics.

	Mean ± S.D	Frequencies/Percentages
Age of patients	28.44 ± 3.95	
Gestational age at delivery	36.40 ± 2.07	
Weight of newborn	2.82 ± 0.47	
Primiparas		10 (13.3%)
Multiparas		65(85.3%)
Delivery at <37 weeks		52(69.3%)
Delivery at > 37 weeks		23(30.07%)
Apgar score <7		11 (14.7%)
Apgar score >7		64(85.3%)

In addition there was statistically significant association between thick placental edge of more than 2cm and birth before 37 weeks (P=0.023). A short cervix of less than 2.5 cm had also statistically significant association with birth before 37 weeks (P=0.022) (Table-II).

**Table II:** Association of thick placental edge and short cervix with preterm birth.

	Delivery at ≥37 weeks	Delivery at ≤ 37 weeks	Row total	P-value
Thin placental edge(<2cm)	12	02	14	0.023
Thick placental edge(>2cm)	29	32	61	
Cervical length less than 2.5cm	10	26	36	0.022
Cervical length more than 2.5cm	21	18	39	

## DISCUSSION

Placental edge thickness correlate negatively with gestation at delivery and also placental thickness of more than 2 cm has significant association with preterm delivery. Cervical length has positive correlation with gestation at delivery and cervical length less than 2.5cm is associated with high risk of preterm birth before 37 weeks.

Zaitoun et al^9^ reported a significant association between thick placental edge of more than 1cm and preterm delivery before 36 weeks. In addition, cervical length less than 30mm ended in emergency cesarean section before 36 weeks in 45.7% cases as compared to 10.5% in cases with cervical length more than 30mm (p value 0.002). Collectively cervical length less than 30mm and lower placental edge thickness more than 10mm had a sensitivity of 83.3% and specificity of 78.45 % in prediction of preterm birth.

Ghi et al^8^ in their prospective cohort study on 59 women suggested that cervical length of 31mm or less increases the chance of emergency cesarean section before 34 weeks in complete placenta previa about 16 times.

The results of current study, regarding thick placental edge and preterm delivery are also supported by a similar prospective study done on 104 patients by Ghourab S et al+0.^13^ They had incorporated shape of the lower placental edge into other transvaginal findings for improving accuracy of predicting preterm delivery and antepartum hemorrhage.

Werlang AC^14^ study suggested that placental edge thickness in complete placenta previa cannot predict outcomes like antepartum hemorrhage and gestational age at delivery. However, it was a retrospective observational study with only 39 patients.

Literature does not give any standard cutoff of thick placental edge. Variations in results among different studies can be due to this lack of standardization. Royal college of obstetricians and gynecologists guidelines on placenta previa has also highlighted the fact that a standardized definition of placental edge thickness should be used before this sign can be used in clinical practice.^5^ Multiparity is a recognized risk factor for placenta previa and our results match with findings of previous studies done to evaluate risk factors for improper placental localization.^15^,^16^

Mean maternal age in the study was 28 years. Farhana et al^17^ also did not find a direct association between advanced maternal age and placenta previa. However, study by Arrul Anne et al concluded maternal age 35 years and above as risk factor for placenta previa.^18^Small sample size may account for this difference. Also getting married at an early age and completing family before age of 35 years can be one of the possible cause.

### Limitations of the Study:

It was a single centre study, with a small sample size and no control group. Larger prospective analytical studies are recommended for stronger evidence of association.

## CONCLUSION

Placental edge thickness has significant negative and cervical length has positive correlation with gestation at delivery. Placental edge thickness of more than 2 cm and cervical length less than 2.5 cm has a significant association with preterm delivery in patients with major placenta previa.

### Authors’ Contribution:

**WS:** Conceived the idea, designed the study, did literature search and drafted manuscript.

**NL:** Did analysis & interpretation of data, edited and finalized manuscript

**GN:** Collected data and its interpretation

**SMK:** Helped in data collection and its analysis

**WS & NL:** Are responsible for the integrity, originality and accuracy of work.
